# Effect of a digital health behaviour change support system on cardiovascular disease risk in a randomized weight loss trial

**DOI:** 10.1038/s41746-026-02747-7

**Published:** 2026-05-11

**Authors:** Eero Turkkila, Heta Merikallio, Markku J. Savolainen, Laura Heikkilä, Harri Oinas-Kukkonen, Tuire Salonurmi, Anna-Maria Teeriniemi, Terhi Jokelainen, Janne Hukkanen

**Affiliations:** 1 Research Unit of Biomedicine and Internal Medicine, University of Oulu, Oulu, Finland; 2 Medical Research Center Oulu, University of Oulu and Oulu University Hospital, Oulu, Finland; 3 Oulu Advanced Research on Service and Information Systems, University of Oulu, Oulu, Finland; 4 Research Unit of Health Sciences and Technology, University of Oulu, Oulu, Finland; 5 Psychiatry, University of Oulu and Oulu University Hospital, Oulu, Finland

**Keywords:** Cardiology, Diseases, Health care, Medical research, Risk factors

## Abstract

Obesity is a risk factor for cardiovascular diseases (CVD). We evaluated a 52-week digital Health Behaviour Change Support System (HBCSS) with a one-year follow-up to treat overweight and Class I obesity. A total of 532 participants (BMI 27–35 kg/m^2^) were randomized into three groups: CBT-based group counselling and self-help guidance (SHG) delivered face-to-face, and usual care. These groups were further divided into HBCSS and non-HBCSS groups. The 10-year CVD risks were assessed using the FINRISK calculator. Baseline median overall CVD risks were similar between groups. In two-group analyses comparing HBCSS and non-HBCSS groups, after 12 months, CVD risks decreased in both groups. However, after 24 months, only the HBCSS group maintained significant reductions in overall risk (−0.40 percentage points, *p* < 0.001). Among participants with obesity, the HBCSS group demonstrated a sustained decrease in overall and coronary artery disease risk, while there were no CVD risk reductions in the non-HBCSS group. However, the differences between HBCSS and non-HBCSS groups were not significant in any analyses conducted, and there were no significant differences in six group analyses between intervention groups. The HBCSS group showed significant reductions in weight, BMI, and waist circumference (at 12 and 24 months) and in LDL cholesterol (at 12 months), compared with controls (*p* < 0.05). The HBCSS results in a decrease in CVD risk factors, which is reflected by a sustained reduction in calculated CVD risks, especially among participants with obesity.

## Introduction

Obesity increases the risk of many diseases, such as type 2 diabetes, cardiovascular diseases, cancer, and death^1–4^. In Europe, cardiovascular diseases (CVD) account for 45% of all deaths^5,6^. The most important factors explaining cardiovascular morbidity are hypertension, smoking, hyperlipidaemia, obesity, and diabetes, explaining more than half of the morbidity^7^. In Finland, around half of people over the age of 30 already have high blood pressure, and nearly one-third are living with obesity^8^.

The risk of cardiovascular diseases can be assessed by combining individual risk factors, such as blood pressure, cholesterol levels, and diabetes, and lifestyle factors, such as smoking. The overall risk may be clinically significant, even if the individual risk factors are only slightly elevated. In Finnish healthcare, the risk of disease in previously healthy patients is estimated using the FINRISK calculator, based on Finnish population cohorts^9^. Other widely used risk calculators include SCORE2 and LIFE-CVD2^10,11^.

The main purpose of obesity treatment is to prevent further weight gain, prevent and treat obesity-related diseases, and improve work and functional capacity and quality of life^12^. Lifestyle intervention can be combined with other forms of treatment (e.g., medication or surgery) if lifestyle intervention alone does not achieve sufficient results^12,13^. Pharmacological treatment is currently one of the most effective conservative treatments; however, there are problems associated with weight regain after discontinuing medication^14^.

Digital interventions have a favourable effect on CVD risk factors, but there is less research on the primary prevention of CVD events^15–17^. The Health Behaviour Change Support System (HBCSS) is a web-based lifestyle change application that utilizes persuasive systems design (PSD) and cognitive behavioural therapy (CBT) methods to treat patients with overweight and obesity. We previously conducted a randomized clinical trial demonstrating the effectiveness of HBCSS in weight loss and reduction of metabolic syndrome, as well as its long-term legacy effect on reducing the need for blood pressure medications^18–20^. The primary objective of this study was to assess the impact of the HBCSS on cardiovascular disease risk using a CVD risk calculator.

## Results

### Study population and baseline characteristics

Out of 532 participants at baseline, 108 dropped out between baseline and 12 months, and an additional 52 dropped out between 12 months and 24 months. Dropout rates did not differ between groups based on BMI category (obesity or overweight) (*p* > 0.05). The results are presented in Supplementary Fig. 2. One participant was excluded from the study due to an erroneous laboratory result to prevent bias from a significant random measurement error. Therefore, a total of 531 participants were analysed.

Table 1 presents the baseline characteristics of the participants. At baseline, the mean (SD) age was 45.9 (10.0) years; 50.7% of participants were male, and 49.3% were female. Among all participants, the median 10-year overall cardiovascular risk was 2.51% (IQR 1.10 to 4.40), coronary artery disease risk was 1.43% (0.55 to 2.86), and stroke risk was 0.97% (0.47 to 1.58). Participants with obesity (BMI 30–35 kg/m^2^) had FINRISK values 1.03, 1.04, and 1.14 times higher at baseline than participants with overweight (BMI 27–29.99 kg/m^2^), respectively. There were no statistically significant differences between HBCSS and non-HBCSS groups at baseline.

**Table 1:** Baseline characteristics of the participants

Variables	HBCSS	No HBCSS	*P* value	Total
	(*n* = 270)	(*n* = 261)		(*n* = 531)
Age, year (SD)	46.1 ± 9.9	45.8 ± 10.1	0.733^a^	45.9 ± 10.0
Sex, *n* (%)				
*Males*	138 (51.1)	131 (50.2)	0.832^b^	269 (50.7)
*Females*	132 (48.9)	130 (49.8)		262 (49.3)
Body weight (kg) (SD)	89.3 ± 10.9	89.7 ± 11.6	0.711^a^	89.5 ± 11.2
Waist circumference (cm) (SD)	100.8 ± 8.2	101.3 ± 7.6	0.583^a^	101.0 ± 7.9
BMI (kg/m^2^) (SD)	30.3 ± 2.1	30.6 ± 2.1	0.232^a^	30.5 ± 2.1
Systolic blood pressure (mmHg) (SD)	130.0 ± 15.8	130.7 ± 16.7	0.661^a^	130.4 ± 16.2
Diastolic blood pressure (mmHg) (SD)	82.5 ± 10.0	82.8 ± 10.5	0.762^a^	82.6 ± 10.3
Glucose (mmol/l) (SD)	5.52 ± 0.59	5.52 ± 0.47	0.982^a^	5.52 ± 0.53
HbA1c (mmol/mol) (SD)	38.70 ± 4.87	38.52 ± 4.35	0.660^a^	38.61 ± 4.61
HDL-c (mmol/l) (SD)	1.48 ± 0.39	1.45 ± 0.33	0.389^a^	1.47 ± 0.36
LDL-c (mmol/l) (SD)	3.64 ± 0.99	3.61 ± 0.91	0.737^a^	3.63 ± 0.95
Total cholesterol (mmol/l) (SD)	5.40 ± 1.00	5.33 ± 0.98	0.400^a^	5.36 ± 0.99
Triglycerides (mmol/l) (SD)	1.47 ± 1.02	1.49 ± 1.11	0.817^a^	1.48 ± 1.06
FINRISK overall 10-year risk (%) (median, IQR)	2.58	2.49	0.616^c^	2.51
	(1.19 to 4.46)	(1.05 to 4.34)		(1.10 to 4.40)
FINRISK coronary artery disease risk (%) (median, IQR)	1.46	1.40	0.593^c^	1.43
	(0.57 to 2.94)	(0.54 to 2.75)		(0.55 to 2.86)
FINRISK stroke risk (%) (median, IQR)	0.96	0.98	0.978^c^	0.97
	(0.49 to 1.51)	(0.44 to 1.62)		(0.47 to 1.58)
**BMI 27–29.9** **kg/m**^**2**^ **(HBCSS** ***n*** = **132, no HBCSS** ***n*** = **121)**				
FINRISK overall 10-year risk (%) (median, IQR)	2.34	2.54	0.400^c^	2.48
	(1.00 to 4.15)	(1.05 to 4.52)		(1.03 to 4.28)
FINRISK coronary artery disease risk (%) (median, IQR)	1.34	1.50	0.416^c^	1.40
	(0.53 to 2.61)	(0.67 to 2.79)		(0.56 to 2.64)
FINRISK stroke risk (%) (median, IQR)	0.91	0.90	0.293^c^	0.90
	(0.40 to 1.37)	(0.43 to 1.78)		(0.41 to 1.56)
**BMI 30–35** **kg/m**^**2**^ **(HBCSS** ***n*** = **138, no HBCSS** ***n*** = **140)**				
FINRISK overall 10-year risk (%) (median, IQR)	2.85	2.41	0.157^c^	2.56
	(1.29 to 5.18)	(1.01 to 4.21)		(1.21 to 4.60)
	*p*^d^ = 0.064	*p*^d^ = 0.584		*p*^d^ = 0.360
FINRISK coronary artery disease risk (%) (median, IQR)	1.49	1.34	0.142^c^	1.45
	(0.70 to 3.41)	(0.47 to 2.74)		(0.55 to 3.14)
	*p*^d^ = 0.122	*p*^d^ = 0.417		*p*^d^ = 0.601
FINRISK stroke risk (%) (median, IQR)	1.08	1.00	0.276^c^	1.03
	(0.59 to 1.71)	(0.44 to 1.57)		(0.50 to 1.58)
	***p***^**d**^ = **0.031**	*p*^d^ = 0.847		*p*^d^ = 0.159

*HBCSS* health behaviour change support system, *BMI* body mass index, *LDL-c* low density lipoprotein cholesterol, *HDL-c* high density lipoprotein cholesterol, *HbA1c* glycated haemoglobin.

^a^Paired samples *t* test.

^b^Pearson Chi-Square Test.

^c^Mann-Whitney U Test.

^d^*p*-value compared with participants with BMI 27–29.9.

### Changes in cardiovascular disease risk factors

The changes (mean (95% CI)) in weight and cardiovascular risk factors after 12 and 24 months are presented in Table 2. After 12 months, the HBCSS group lost more weight (−2.30 kg (−2.91 to −1.68), *p* < 0.001) compared with the non-HBCSS group (−0.76 kg (−1.39 to −0.13), *p* = 0.019), with a significant difference (*p* < 0.001) between groups. Similar patterns were observed for BMI (−0.76 vs. −0.27 kg/m², *p* < 0.001 between groups) and waist circumference (−2.42 vs. −0.68 cm, *p* < 0.001 between groups). Although both groups showed significant reductions in systolic and diastolic blood pressure, the differences between the groups were not statistically significant (*p* = 0.676 and *p* = 0.717, respectively). LDL cholesterol level was significantly reduced in the HBCSS group (−0.17 mmol/l (−0.26 to −0.09), *p* < 0.001), compared with the non-HBCSS group (−0.01 mmol/l (−0.10 to 0.08), *p* = 0.824), with *p* = 0.011 between groups. There was no difference between the groups for other cardiovascular risk factors (Table 2).

**Table 2:** Mean difference (MD) estimates (95% CI) of the interaction term (group x time) for change in cardiovascular risk factors after 12 and 24 months

	Change after intervention (12 months)					Change after intervention (24 months)						
Variables	HBCSS	*P* value	No HBCSS	*P* Value	*P* value	HBCSS	*P* value	*P* value	No HBCSS	*P* Value	*P* value	*P* value
	(*n* = 270)	a	(*n* = 261)	a	b	(*n* = 270)	c	d	(*n* = 261)	c	d	b
Weight change (kg)	**−2.30**	**< 0.001**	**−0.76**	**0.019**	**< 0.001**	**−1.74**	**< 0.001**	0.511	−0.41	0.751	0.705	**0.012**
	**(–2.91 to –1.68)**		**(–1.39 to –0.13)**			**(–2.56 to –0.91)**			(–1.27 to 0.45)			
BMI (kg/m^2^)	**−0.76**	**< 0.001**	**−0.27**	**0.013**	**< 0.001**	**−0.64**	**< 0.001**	0.936	−0.14	0.714	0.715	**0.005**
	**(–0.97 to –0.50)**		**(–0.49 to –0.06)**			**(–0.92 to –0.37)**			(–0.43 to 0.15)			
Waist circumference (cm)	**−2.42**	**< 0.001**	**−0.68**	**0.040**	**< 0.001**	**−2.92**	**< 0.001**	0.459	**−1.32**	**< 0.001**	0.294	**0.005**
	**(–3.05 to –1.80)**		**(–1.33 to –0.03)**			**(–3.75 to –2.08)**			**(–2.19 to –0.44)**			
Systolic blood pressure (mmHg)	**−5.71**	**< 0.001**	**−6.30**	**< 0.001**	0.676	**−5.36**	**< 0.001**	1.000	**−3.95**	**< 0.001**	0.132	0.327
	**(–7.66 to –3.75)**		**(–8.31 to –4.29)**			**(–7.69 to –3.03)**			**(–6.37 to –1.53)**			
Diastolic blood pressure (mmHg)	**−4.09**	**< 0.001**	**−3.81**	**< 0.001**	0.717	**−2.94**	**< 0.001**	0.144	**−2.14**	**< 0.001**	**0.016**	0.287
	**(–5.14 to –3.04)**		**(–4.89 to –2.73)**			**(–4.28 to –1.60)**			**(–3.53 to –0.75)**			
Glucose (mmol/l)	−0.04	0.108	–0.01	0.892	0.520	**−0.12**	**0.012**	0.099	–0.01	1.000	1.000	0.123
	(–0.11 to 0.03)		(–0.08 to 0.07)			**(–0.21 to –0.02)**			(–0.11 to 0.09)			
HbA1c (mmol/mol)	**−0.61**	**0.011**	**–1.04**	**< 0.001**	0.215	**−2.21**	**< 0.001**	**< 0.001**	**−2.13**	**< 0.001**	**< 0.001**	0.955
	**(–1.08 to –0.14)**		**(–1.52 to –0.56)**			**(–2.72 to –1.73)**			**(–2.64 to –1.62)**			
HDL-c (mmol/l)	−0.01	0.734	**–0.03**	**0.030**	0.187	0.03	0.192	0.082	−0.00	1.000	0.439	0.144
	(–0.03 to 0.02)		**(–0.06 to –0.00)**			(–0.01 to 0.07)			(–0.05 to 0.04)			
LDL-c (mmol/l)	**−0.17**	**< 0.001**	−0.01	0.824	**0.011**	−0.05	1.000	**0.020**	−0.07	0.511	0.713	0.611
	**(–0.26 to –0.09)**		(–0.10 to 0.08)			(–0.17 to 0.08)			(–0.20 to 0.06)			
Total cholesterol (mmol/l)	**–0.14**	**0.003**	−0.04	0.415	0.140	−0.10	0.173	1.000	**−0.16**	**0.014**	0.099	0.367
	**(–0.23 to –0.05)**		(–0.13 to 0.06)			(–0.23 to 0.03)			**(–0.30 to –0.02)**			
Triglycerides (mmol/l)	**–0.17**	**< 0.001**	**−0.15**	**0.005**	0.728	**−0.16**	**0.020**	0.713	−0.14	0.073	1.000	0.813
	**(–0.27 to –0.07)**		**(–0.25 to –0.05)**			**(–0.31 to –0.02)**			(–0.29 to 0.01)			

Data are presented as means (95% CI). Statistically significant changes are presented in bold. The data were analysed using a linear mixed model.

*HBCSS* health behaviour change support system, *BMI* body mass index, *LDL-c* low density lipoprotein cholesterol, *HDL-c* high density lipoprotein cholesterol, *HbA1c* glycated haemoglobin

*p*^a^ = *p*-value within group between baseline and 12 months.

*p*^b^ = *p*-value between groups.

*p*^c^ = *p*-value within group between baseline and 24 months.

*p*^d^ = *p*-value within group between 12 months and 24 months.

At 24 months, many improvements observed at 12 months were sustained, especially in the HBCSS group. In the HBCSS group, weight, BMI, and waist circumference did not change significantly between 12 and 24 months (*p* = 0.705, *p* = 0.715 and *p* = 0.294), with measured values at 24 months −1.74 kg, −0.64 kg/m^2^ and −2.92 cm, respectively; all *p* < 0.001 compared with baseline (with *p* = 0.012, *p* = 0.005, and *p* = 0.005, between groups, respectively). Systolic and diastolic blood pressure remained significantly reduced in both groups. The reduction in LDL cholesterol was not sustained at 24 months in the HBCSS group.

### Changes in calculated cardiovascular disease risks

**Fig. 1:**
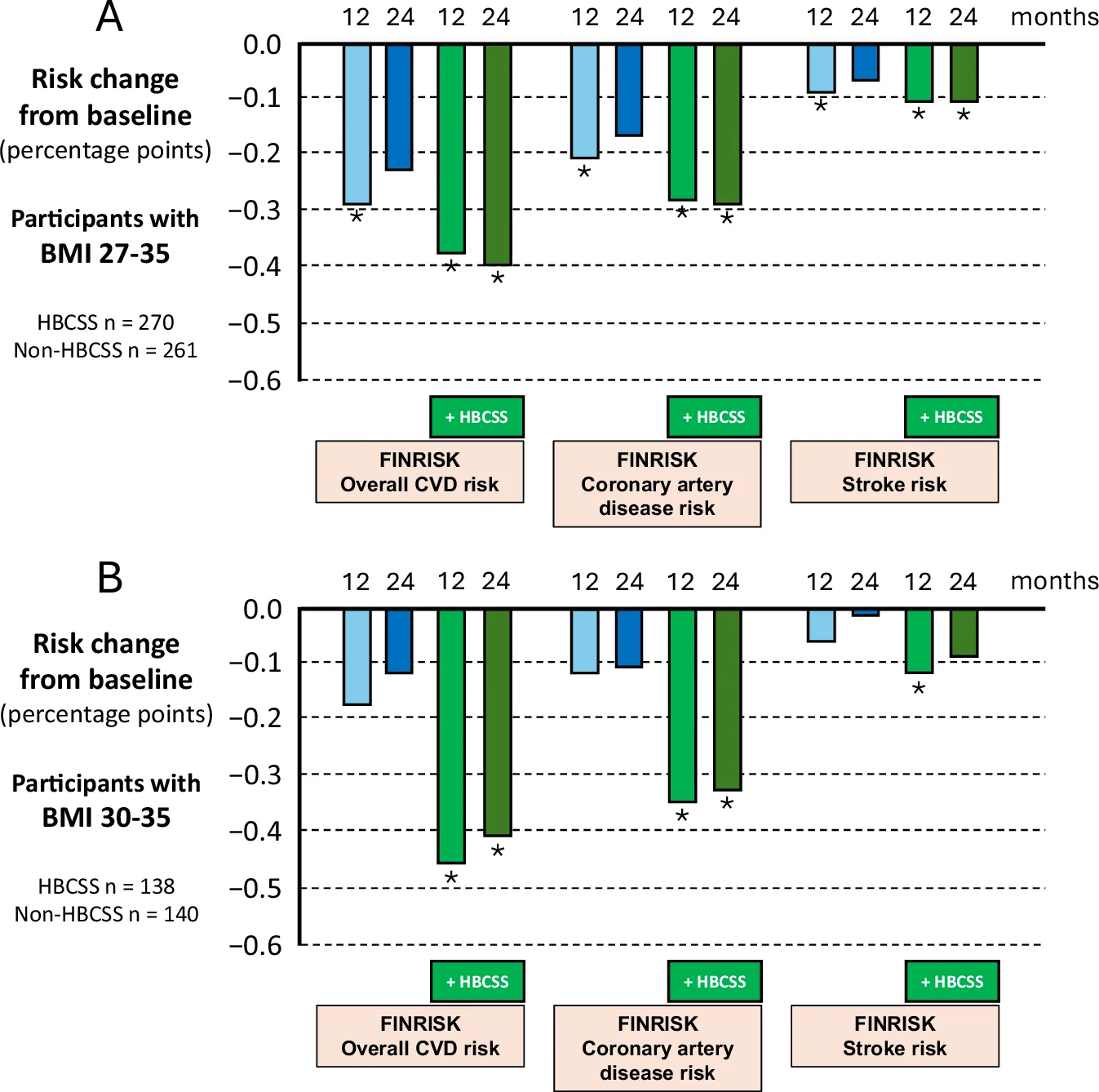
Changes in 10-year cardiovascular disease risks assessed with the FINRISK calculator after 12 and 24 months. Panel **A** represents participants with a BMI of 27–35 kg/m^2^; panel **B** represents participants with a BMI of 30–35 kg/m^2^. *p < 0.05, in comparison with baseline. Risk changes are presented as percentage points. Blue bars, non-HBCSS groups; green bars, HBCSS groups. Lighter colours represent the change at 12 months and darker colours at 24 months.

Among all participants, both groups demonstrated significant reductions (in percentage points) in overall 10-year risk, coronary artery disease risk, and stroke risk at 12 months (Fig. 1, Supplementary Table 1). At 24 months, only the HBCSS group maintained significant reductions in overall 10-year risk (1.74-fold), coronary artery disease risk (1.71-fold), and stroke risk (1.57-fold) compared with the non-HBCSS group, which showed no significant reductions. However, the differences between groups were not statistically significant.

Among participants with obesity (BMI 30–35), at 12 months, the reductions in overall 10-year risk, coronary artery disease risk, and stroke risk were significant only in the HBCSS group (Fig. 1, Supplementary Tables 2). However, the differences between the groups did not reach statistical significance (overall CVD risk *p* = 0.073, CAD risk *p* = 0.067, and stroke risk *p* = 0.248). At 24 months, the reduction in the HBCSS group remained significant in the overall and coronary artery disease risk, with no statistically significant differences between the groups.

The changes were also analysed among the six groups of the original study design, and the results were heterogeneous. There were no significant differences between groups or compared with the control arm for the overall 10-year CVD-risk, CAD risk, or stroke risk (Supplementary Table 3). Also, the results of the sensitivity analyses were similar. Those results are not shown. In addition, no statistically significant difference was found between the groups (*p* > 0.05) in analyses examining the usage data (Supplementary Fig. 3).

## Discussion

We have previously demonstrated that the HBCSS intervention effectively reduces weight for up to two years, supports long-term weight management, and reduces the need for antihypertensive medications for at least four years after the intervention^18,19^. In addition, the HBCSS effect in reducing the prevalence of metabolic syndrome has been previously described^20^. Here, we demonstrate that both HBCSS and non-HBCSS groups demonstrated decreased CVD risks in a two-group analysis after 12 months. After 24 months, only the HBCSS group maintained significant reductions in CVD risks. However, the differences between the HBCSS and non-HBCSS groups were not statistically significant.

When examining individual risk factors, the HBCSS intervention significantly reduced weight, waist circumference, and LDL cholesterol compared with the control group. However, as the overall risk of cardiovascular disease is a combination of individual risk factors, it is essential to look beyond the individual risk factors to the whole^21^. Additionally, when using a risk calculator, patients can more easily understand the estimated CVD risk.

FINRISK reflects the 10-year risk of developing and dying from specific CVD events. A risk level of <5% is considered low, and >10% is considered high^9^. The results obtained with the FINRISK calculator closely correlate with the actual prevalence, as the original FINRISK study found that in a low-risk group (< 5%), the disease prevalence was 1.7% among men and 1.5% among women, respectively^9^. The study population consisted primarily of middle-aged, apparently healthy individuals without known atherosclerotic disease. Therefore, the absolute risk percentages obtained at baseline and at 12 and 24 months are relatively low, compared with the Finnish population average^22^. However, even in such a healthy study population, the risk changes are significant, particularly among participants with obesity. This suggests that the higher the patient’s baseline risk, the greater the absolute benefit of the HBCSS intervention. From an economic perspective, preventing a single myocardial infarction saves an average of more than €20,000 in Finland during the first year alone^23^.

A recently published, extensive cohort study demonstrates that weight loss through midlife lifestyle changes is beneficial for long-term survival and mortality^24^. Obesity has been shown to significantly increase the risk of coronary heart disease and stroke, as well as other cardiometabolic and cardiovascular diseases, including atrial fibrillation^25,26^. HBCSS aims to guide individuals in making long-term lifestyle changes by altering thoughts, feelings, and behaviours, leading to weight loss. The HBCSS also aims to raise awareness about the impact of one’s lifestyle, feelings, and environment on their weight, health, and well-being. In addition to weight management, changes in eating behaviour can also lead to healthier eating habits. FINRISK assesses CVD risk based on individual-defined risk factors. Still, the actual risk of cardiovascular disease may also be influenced by well-being-related factors and biomarkers that cannot be assessed with FINRISK but can be influenced by HBCSS^27,28^.

Risk calculators are most applicable to the population on which they are based. As the trial was performed in Finland, it was reasonable to use the FINRISK calculator. It is also recognized by the Finnish National Current Care Guidelines. One study compared the previous version of the FINRISK calculator with other CVD risk calculators, including SCORE and Framingham^22^. The FINRISK and the Framingham model account for both mortality and morbidity, whereas the original SCORE model focused solely on mortality. The current SCORE2 provides a composite of cardiovascular mortality, non-fatal myocardial infarction, and non-fatal stroke. The Framingham morbidity data also include milder coronary cases, whereas the FINRISK model considers only major coronary events as outcomes. Therefore, although the results obtained with the different calculators are mostly similar, the various definitions of the end events yield slightly different risk scores^22^.

The original randomized trial design, along with the relatively large number of participants and the measurements controlled by healthcare professionals, supports the reliability of the results in this study. Confounding factors were minimized; however, the lack of more detailed family history and cardiovascular risk data may affect the baseline risk level. It should be acknowledged that there is an overlap between weight loss and the variables that constitute the CVD risk scores. Therefore, changes in the FINRISK score should be interpreted as a composite measure of the metabolic benefits of weight loss. As the inclusion criteria for this trial were BMI 27 to 35, the results reflect changes in the risk levels associated with overweight and mild, class I obesity.

In addition to offering a scalable solution for weight loss, our findings suggest that the HBCSS may lower the risk of cardiovascular diseases, especially in patients living with class I obesity. Further research is warranted to demonstrate the effectiveness of the HBCSS on the occurrence of actual CVD events.

## Methods

### Trial design

The Prevent Metabolic Syndrome (PrevMetSyn) was designed as a randomized intervention trial that included a population-based sample of participants with overweight or class I obesity (BMI 27–35 kg/m^2^). The procedures in this trial followed the guidelines of the Declaration of Helsinki^29^. The study design is registered under ClinicalTrials.gov with registration ID NCT01959763. The Ethics Committee of the North Ostrobothnia Hospital District, Oulu, Finland, has approved the PrevMetSyn study with decision 29/2012 (March 26, 2012) and amendment statements dated November 23, 2012, February 18, 2013, and May 7, 2018.

The research data is stored at Oulu University Hospital, and access is limited to members of the research group. Individual identities are pseudonymized, and data are processed using research ID numbers. The participants gave written consent and had the option to withdraw from the study at any time. The principles of Good Clinical Practice were adhered to. The storage and use of software and personal data comply with the General Data Protection Regulation (GDPR).

### Participants

Participants for this trial were recruited by sending an invitation letter to 11,400 people aged 20–60 years living in Oulu, Finland, according to the Population Registry. A total of 1065 people responded to the invitation and were assessed for eligibility. After exclusion, 532 volunteers with a BMI between 27 and 35 kg/m^2^, internet access, and no treatment for obesity or contraindications to weight loss were included. Exclusion criteria included uncontrolled health factors, such as abnormal laboratory values (thyroid, kidney, and liver function tests), or clinically significant illness that contraindicated weight loss or physical activity^18^.

### Intervention and treatment arms

**Fig. 2:**
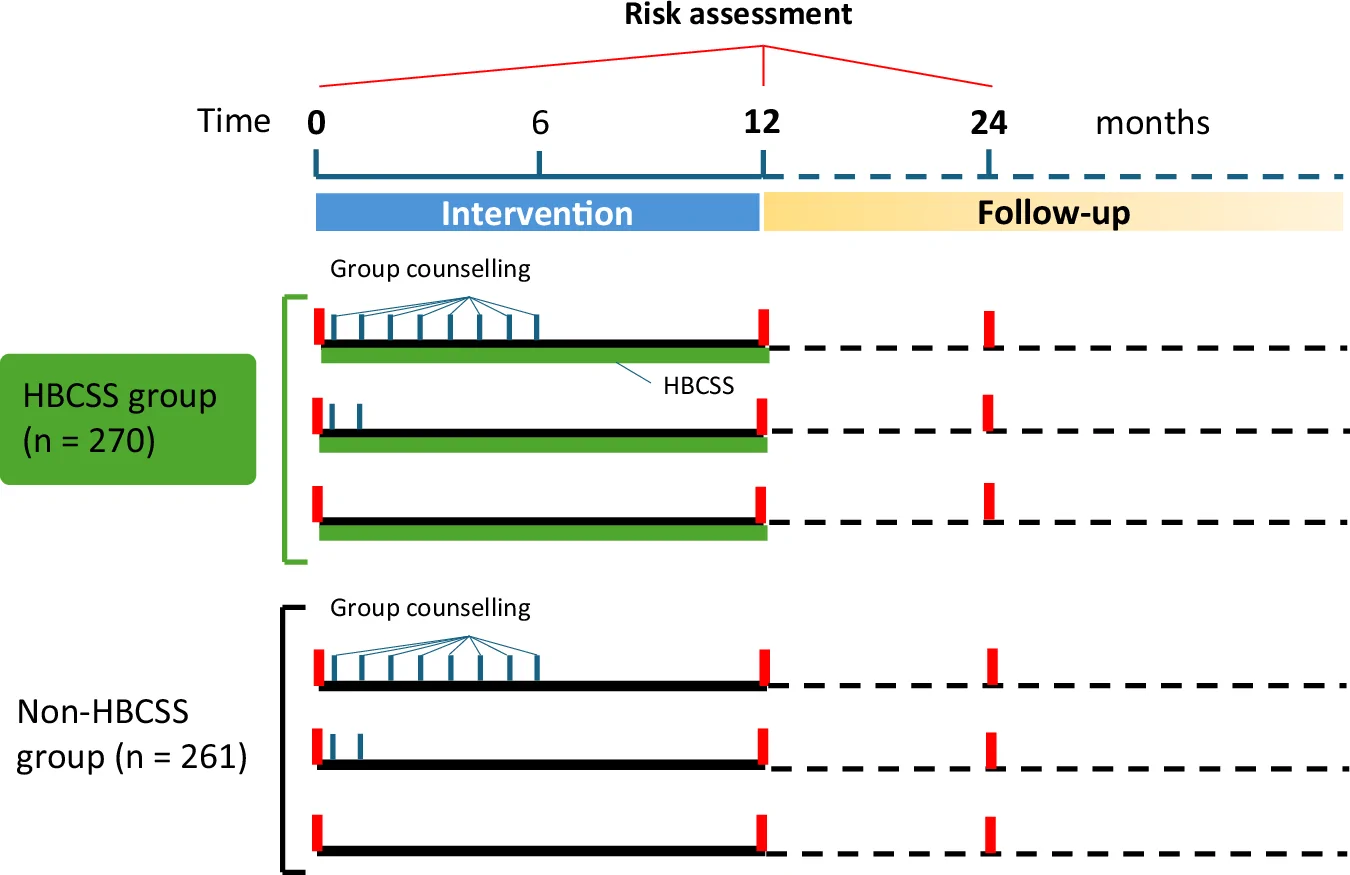
The original six treatment arms were combined into two groups analysed in this paper. Cardiovascular disease risks were assessed at baseline, 12 months, and 24 months. HBCSS, health behaviour change support system.

The 532 enrolled participants were assigned to two groups that received different intensities of face-to-face group counselling or to a control arm. Each of these three groups was then further randomized into web-based HBCSS users and non-users (Supplementary Fig. 1). This paper focuses on the HBCSS intervention, and the groups are categorized into two groups: those with and those without HBCSS (Fig. 2).

Face-to-face group counselling included cognitive-behavioural therapy (CBT) based counselling and self-help guidance (SHG). CBT-based group counselling included eight 90-min group sessions with two clinical nutritionists per group, aimed at helping participants recognize thoughts, feelings, and behaviours related to eating and weight, and to change them to support weight management and weight loss. The groups had eight to nine participants. Mindfulness exercises, psychoeducation, and individual, pair, and group exercises were used during the sessions, along with home tasks. CBT methods included techniques to identify and cope with dysfunctional thoughts, to recognize high-risk situations related to uncontrolled or unplanned eating, and problem-solving skills. Self-monitoring and planning were utilized to follow changes. SHG group counselling included two 90-min sessions based on the Elvira counselling model, utilizing the constructive learning theory and stage of change model^30,31^. SHG group sessions were led by a registered nurse and aimed to improve self-help skills to change lifestyle and eating habits. The control group received information about weight management and metabolic syndrome, which is the usual care provided in primary healthcare.

The HBCSS was a web-based information system designed for this trial, based on PSD combined with CBT methods. The HBCSS was a stand-alone 52-week programme designed to guide users toward a healthier lifestyle, improved eating behaviours, and weight loss. Self-monitoring was an important part of the system and included tools to track lifestyle progress, such as weight, meal rhythm, and physical activity. The HBCSS provided weekly information on health, weight loss, and exercises to recognize, change, and cope with eating, weight, and lifestyle-related issues. The software features of the PSD were designed to support users in performing their primary tasks, enhance computer-human interaction, and increase perceived credibility and social influence^32,33^. Participants interacted with the web-based information system weekly throughout the 52-week intervention. The content of the HBCSS was based on current knowledge of eating psychology, nutrition, and obesity treatment.

The original trial design included baseline and 12- and 24-month visits. Later, an additional 60-month visit was added^19^. Height, weight, and waist circumference were measured, and blood samples were collected by a nurse at the research centre of Oulu University Hospital, Finland. The weight change analysis, as outlined in the primary protocol, along with information on the treatment arms, sample size calculations, and randomization, has been presented in previous publications^18–20^.

### Risk assessment

We assessed the overall risk of cardiovascular diseases at baseline and at 12 and 24 months after the intervention period using the Finnish CVD risk calculator, FINRISK. The FINRISK is primarily designed to help clinicians assess an apparently healthy patient’s overall 10-year cardiovascular risk, which can be challenging^9^. It is actively used in Finland and has been utilized nationwide since 2007. The FINRISK calculator is based on extensive data from Finnish population surveys over the decades, including risk factor and morbidity data. It calculates a patient’s 10-year risk (%) for coronary artery disease, stroke, and an overall risk, which is the risk of having either of the two.

The FINRISK considers the patient’s sex, age, total cholesterol (mmol/l), high-density lipoprotein (HDL) cholesterol (mmol/l), smoking status, diabetes status, systolic blood pressure (mmHg), and family history of myocardial infarction and stroke. In our data, information on family history was not available. There is no generally accepted coefficient to describe the mean history of cardiovascular risk at the mean age of our study population. As this study examines intra-individual changes and family history is not a changing risk factor in this context, the coefficient was set to 0 in the formula.

The FINRISK calculator is designed for risk calculations for individuals aged 30 to 74. Although most participants fell within this age range, a few were under 30. They were included in the analysis because the change in risk is the study’s primary analysis. Additionally, age was fixed at the baseline when analysing changes from baseline to 12 and 24 months to minimize confounding variables that could affect the CVD risk scores.

### Statistical methods

The primary outcome was the absolute change (in percentage points) in CVD risk between baseline and 12 and 24 months. Changes in risks between HBCSS and non-HBCSS groups were analysed among all participants using the intention-to-treat (ITT) principle.

A linear mixed-effects model (LMM) was used to assess changes in FINRISK values from baseline to 12 and 24 months. LMM was also used to analyse changes in cardiovascular risk factors over the follow-up period. Group and time were used as fixed effects^34^. The model also included a group and time interaction term to evaluate differences in mean change over time between groups, adjusting for age and sex. Missing data were assumed to be missing at random. As the LMM allows analysis with unbalanced datasets, no imputation was needed for the primary analyses. The analyses were also conducted using an LMM with imputed values based on the return-to-baseline (RTB) method, and with completers only, as sensitivity analyses. Changes in FINRISK values from baseline to 12 and 24 months across six groups of the original study design were also analysed as a sensitivity analysis.

Differences in baseline characteristics between groups were analysed using Paired Samples *t* tests and Chi-square tests. Baseline CVD risk differences between groups were analysed using the Mann-Whitney U test.

IBM SPSS Statistics version 26.0 (IBM Corp., Armonk, NY, USA) was used for the analyses. *p*-values less than 0.05 were considered statistically significant.

## Supplementary information

CONSORT checklist

Supplementary Information

## Data Availability

The data supporting this study's findings are available from the corresponding author upon request. The data are not publicly available due to privacy or ethical restrictions.
