# Author Correction: Effect of a digital health behaviour change support system on cardiovascular disease risk in a randomized weight loss trial

**DOI:** 10.1038/s41746-026-03190-4

**Published:** 2026-09-03

**Authors:** Eero Turkkila, Heta Merikallio, Markku J. Savolainen, Laura Heikkilä, Harri Oinas-Kukkonen, Tuire Salonurmi, Anna-Maria Teeriniemi, Terhi Jokelainen, Janne Hukkanen

**Affiliations:** 1 Research Unit of Biomedicine and Internal Medicine, University of Oulu, Oulu, Finland; 2 Medical Research Center Oulu, University of Oulu and Oulu University Hospital, Oulu, Finland; 3 Oulu Advanced Research on Service and Information Systems, University of Oulu, Oulu, Finland; 4 Research Unit of Health Sciences and Technology, University of Oulu, Oulu, Finland; 5 Psychiatry, University of Oulu and Oulu University Hospital, Oulu, Finland

**Keywords:** Cardiology, Diseases, Health care, Medical research, Risk factors

Correction to: *npj Digital Medicine* https://doi.org/10.1038/s41746-026-02747-7, published online 11 May 2026

The reported value in the Abstract has been corrected from ‘−0.40%’ to ‘−0.40 percentage points.’

