# Supplementary material for: Effect of a digital health behaviour change support system on cardiovascular disease risk in a randomized weight loss trial
Source: NPJ Digit Med. 2026 May 11;9:560. doi: 10.1038/s41746-026-02747-7 (PMC13385364; doi:10.1038/s41746-026-02747-7)
Supplement: Supplementary file 1 — CONSORT checklist [file 41746_2026_2747_MOESM1_ESM.pdf]

SUPPLEMENTARY INFORMATION

Effect of a Digital Health Behaviour Change Support System on Cardiovascular Disease Risk in a Randomized Weight Loss Trial

Eero Turkkila, Heta Merikallio, Markku J. Savolainen, Laura Heikkilä, Harri Oinas-Kukkonen, Tuire Salonurmi, Anna-Maria Teeriniemi, Terhi Jokelainen, Janne Hukkanen

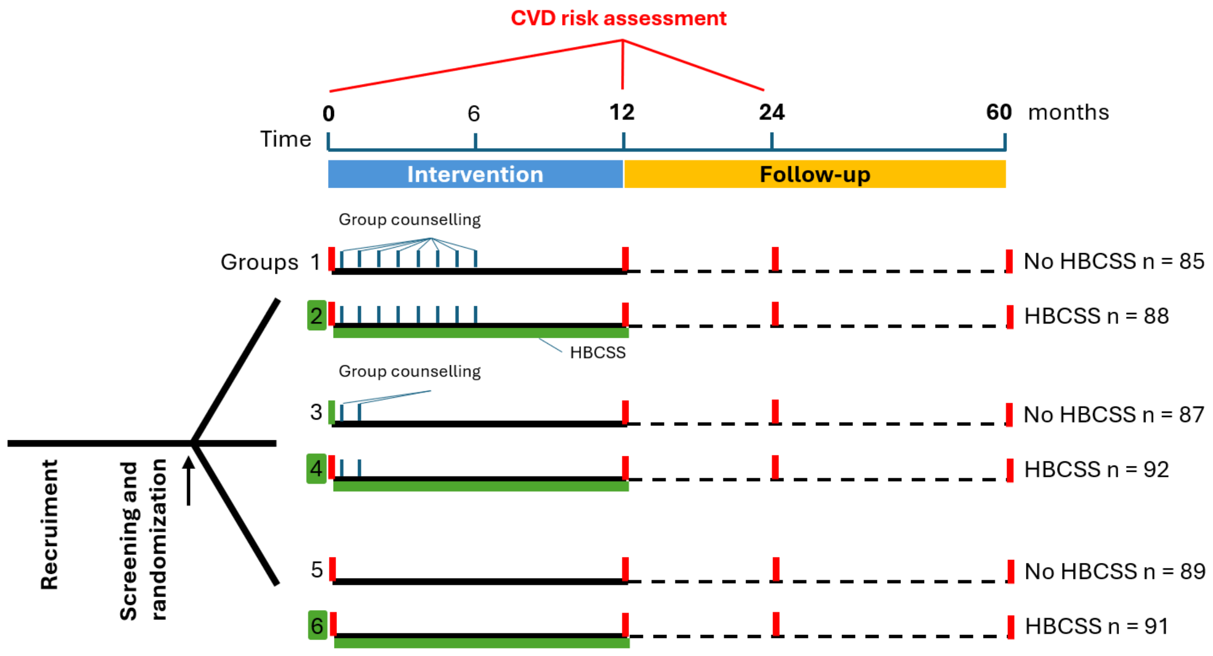

Supplementary Figure 1. Design of the original trial with six treatment arms and 532 participants.
Groups 1 and 2 represent groups receiving cognitive behavioural therapy (CBT) based counselling, with or without HBCSS. Groups 3 and 4 represent groups receiving self-help guidance (SHG) group counselling, with or without HBCSS, and 5 and 6 represent the control arms, respectively.

HBCSS, a web-based health behaviour change support system

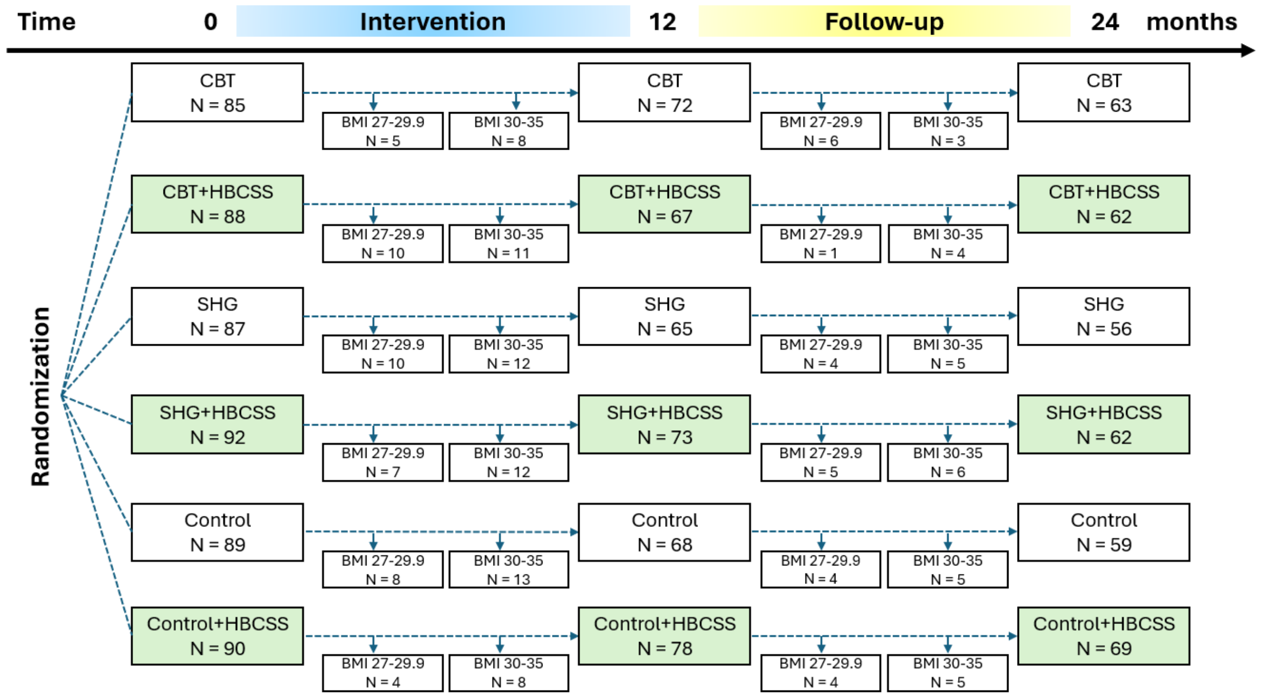

Supplementary Figure 2. Flowchart of the participants of this study (*n* = 531). Boxes between 0 and 12 months, and 12 and 24 months represent dropouts in each group, divided by BMI. There were no significant differences between groups in the number of dropouts ($p > 0.05$). Green represents groups with HBCSS counselling.

CBT, cognitive behavioural therapy (CBT) based group counselling; SHG, self-help guidance (SHG) group counselling; HBCSS, a web-based health behaviour change support system.

Supplementary Table 1. Mean difference (MD) estimates of the interaction term (group x time) for the change in risk calculated with FINRISK (percentage points) after 12 and 24 months in the whole study population.

Group	Change after 12 months		Change after 24 months		
**BMI 27-35**	**MD (95 % CI)**	***p*-value[a]**	**MD (95 % CI)**	***p*-value[c]**	***p*-value[d]**
**Overall 10-year risk**					
HBCSS (n = 270)	**–0.37 (–0.58 to –0.17)**	**<0.001**	**–0.40 (–0.63 to –0.16)**	**<0.001**	1.000
No HBCSS (n = 261)	**–0.29 (–0.51 to –0.08)**	**0.003**	–0.23 (–0.48 to 0.01)	0.070	1.000
	p^b = 0.480		p^b = 0.269		
**Coronary artery disease risk**					
HBCSS (n = 270)	**–0.28 (–0.45 to –0.10)**	**<0.001**	**–0.29 (–0.49 to –0.10)**	**0.001**	1.000
No HBCSS (n = 261)	**–0.21 (–0.39 to –0.03)**	**0.013**	–0.17 (–0.38 to 0.03)	0.125	1.000
	p^b = 0.497		p^b = 0.315		
**Stroke risk**					
HBCSS (n = 270)	**–0.11 (–0.17 to –0.05)**	**<0.001**	**–0.11 (–0.18 to –0.04)**	**<0.001**	1.000
No HBCSS (n = 261)	**–0.09 (–0.15 to –0.03)**	**<0.001**	–0.07 (–0.14 to 0.00)	0.073	1.000
	p^b = 0.617		p^b = 0.293		

Data analyzed with Linear Mixed Model
CI: Confidence interval
HBCSS, a web-based health behaviour change support system

p^a= *p*-value within group between baseline and 12 months
p^b= *p*-value between groups
p^c= *p*-value within group between baseline and 24 months
p^d= *p*-value within group between 12 and 24 months

Supplementary Table 2. Mean difference (MD) estimates of the interaction term (group x time) for the change in risk calculated with FINRISK (percentage points) after 12 and 24 months among participants living with obesity.

Group	Change after 12 months		Change after 24 months		
**BMI 30-35**	**MD (95 % CI)**	***p*-value[a]**	**MD (95 % CI)**	***p*-value[c]**	***p*-value[d]**
**Overall 10-year risk**					
HBCSS (n = 138)	**–0.46 (–0.73 to –0.19)**	**<0.001**	**–0.41 (–0.79 to –0.04)**	**0.027**	1.000
No HBCSS (n = 140)	–0.18 (–0.45 to 0.09)	0.322	–0.12 (–0.49 to 0.26)	1.000	1.000
	p = 0.073		p^b = 0.187		
**Coronary artery disease risk**					
HBCSS (n = 138)	**–0.35 (–0.56 to –0.13)**	**<0.001**	**–0.33 (–0.63 to –0.03)**	**0.024**	1.000
No HBCSS (n = 140)	–0.12 (–0.33 to 0.09)	0.500	–0.11 (–0.41 to 0.18)	1.000	1.000
	p = 0.067		p^b = 0.212		
**Stroke risk**					
HBCSS (n = 138)	**–0.12 (–0.21 to –0.04)**	**0.002**	–0.09 (–0.21 to 0.03)	0.191	1.000
No HBCSS (n = 140)	–0.06 (–0.15 to 0.02)	0.222	–0.01 (–0.12 to 0.11)	1.000	0.736
	p = 0.248		p^b = 0.244		

Data analyzed with Linear Mixed Model
CI: Confidence interval
HBCSS, a web-based health behaviour change support system

p^a= *p*-value within group between baseline and 12 months
p^b= *p*-value between groups
p^c= *p*-value within group between baseline and 24 months
p^d= *p*-value within group between 12 and 24 months

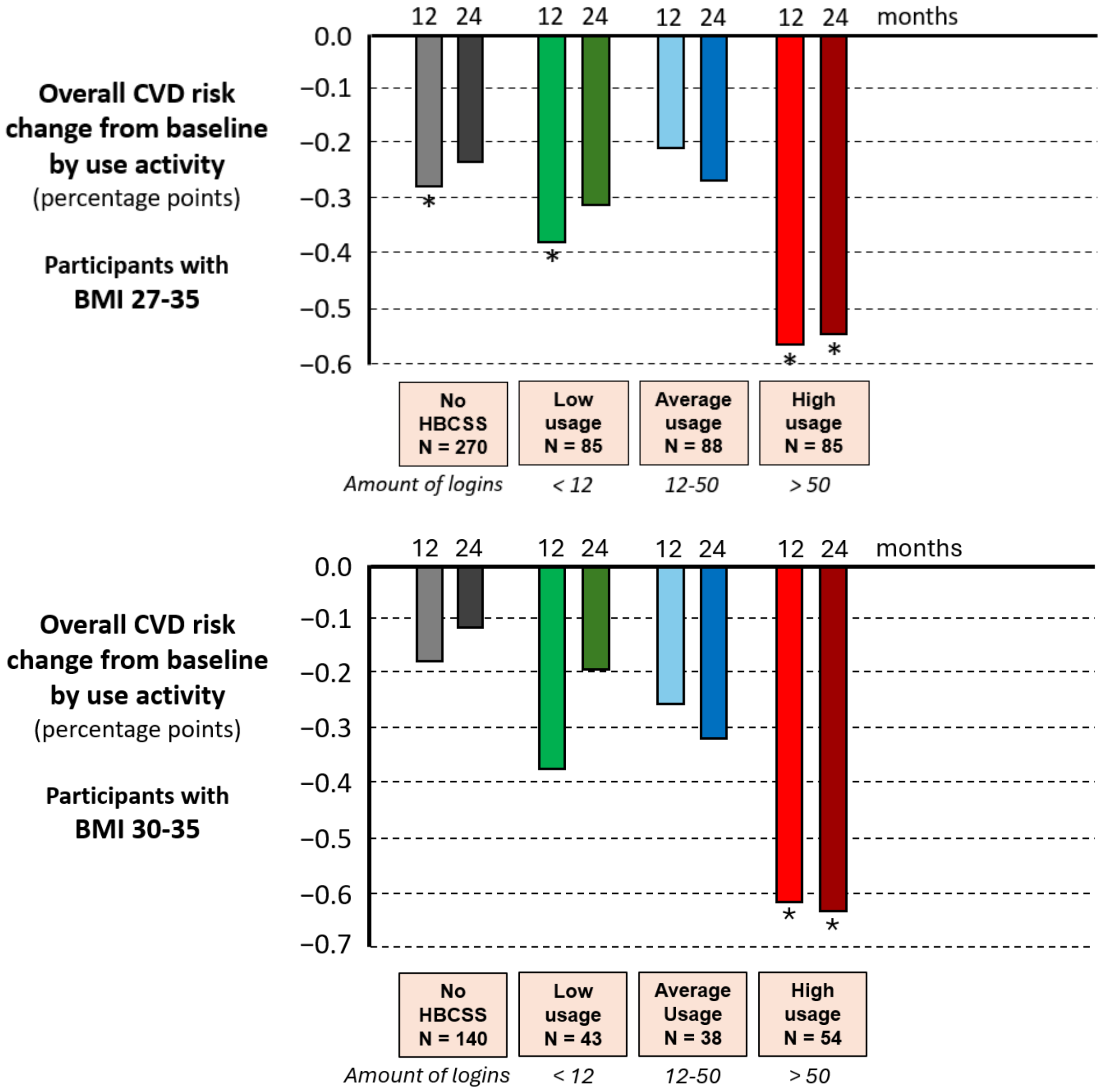

Supplementary Figure 3. Overall 10-year CVD risk change by HBCSS usage. There was no significance between groups.

HBCSS, a web-based health behaviour change support system
*p < 0.05, in comparison with baseline.

Supplementary Table 3. Mean difference (MD) estimates of the interaction term (group x time) for the change in risk calculated with FINRISK (percentage points) after 12 and 24 months in the whole study population in six groups.

**Group**	**Change after 12 months**		**Change after 24 months**		
**BMI 27-35**	**MD (95 % CI)**	***p*-value[a]**	**MD (95 % CI)**	***p*-value[c]**	***p*-value[d]**
**Overall 10-year risk**					
CBT (N = 85)	**–0.38 (–0.74 to –0.03)**	**0.031**	–0.22 (–0.64 to 0.20)	0.627	0.859
CBT+HBCSS (N = 88)	**–0.45 (–0.82 to –0.08)**	**0.010**	–0.23 (–0.65 to 0.20)	0.605	0.440
SHG (N = 87)	**–0.45 (–0.82 to –0.07)**	**0.013**	–0.20 (–0.64 to 0.24)	0.815	0.635
SHG+HBCSS (N = 92)	**–0.45 (–0.80 to –0.10)**	**0.007**	**–0.45 (–0.87 to –0.03)**	**0.030**	0.369
Control (N = 89)	–0.06 (–0.42 to 0.31)	1.000	–0.28 (–0.71 to 0.15)	0.344	0.477
Control+HBCSS (N = 90)	–0.25 (–0.59 to 0.10)	0.262	**–0.50 (–0.90 to –0.10)**	**0.008**	0.093
	p^b = 0.299		p^b = 0.207		
**Coronary artery disease risk**					
CBT (N = 85)	–0.26 (–0.56 to 0.04)	0.104	–0.16 (–0.51 to 0.19)	0.807	1.000
CBT+HBCSS (N = 88)	**–0.31 (–0.62 to –0.00)**	**0.048**	–0.15 (–0.50 to 0.20)	0.919	0.581
SHG (N = 87)	**–0.38 (–0.69 to –0.07)**	**0.012**	–0.17 (–0.53 to 0.19)	0.772	0.337
SHG+HBCSS (N = 92)	**–0.35 (–0.65 to –0.06)**	**0.013**	–0.33 (–0.67 to 0.20)	0.071	0.321
Control (N = 89)	0.00 (–0.30 to 0.31)	1.000	–0.19 (–0.54 to 0.16)	0.593	0.374
Control+HBCSS (N = 90)	**–0.35 (–0.65 to –0.06)**	**0.013**	–0.33 (–0.67 to 0.02)	0.071	1.000
	p^b = 0.235		p^b = 0.162		
**Stroke risk**					
CBT (N = 85)	**–0.13 (–0.23 to –0.03)**	**0.005**	–0.07 (–0.19 to 0.06)	0.587	0.562
CBT+HBCSS (N = 88)	**–0.15 (–0.26 to –0.05)**	**0.001**	–0.08 (–0.20 to 0.05)	0.427	0.345
SHG (N = 87)	–0.08 (–0.19 to 0.02)	0.181	–0.03 (–0.16 to 0.10)	1.000	0.887
SHG+HBCSS (N = 92)	**–0.11 (–0.21 to –0.01)**	**0.021**	**–0.14 (–0.26 to –0.02)**	**0.016**	1.000
Control (N = 89)	–0.06 (–0.17 to 0.04)	0.401	–0.11 (–0.24 to 0.02)	0.107	1.000
Control+HBCSS (N = 90)	–0.07 (–0.17 to 0.02)	0.205	**–0.12 (–0.24 to –0.00)**	**0.037**	0.898
	p^b = 0.591		p^b = 0.495		

Data analyzed with Linear Mixed Model
CI: Confidence interval HBCSS, a web-based health behaviour change support system

p^a= *p*-value within group between baseline and 12 months
p^b= *p*-value between groups
p^c= *p*-value within group between baseline and 24 months
p^d= *p*-value within group between 12 and 24 months
